# Overview of Pancreatic Cancer Epidemiology in Europe and Recommendations for Screening in High-Risk Populations

**DOI:** 10.3390/cancers15143634

**Published:** 2023-07-15

**Authors:** Olga Partyka, Monika Pajewska, Daria Kwaśniewska, Aleksandra Czerw, Andrzej Deptała, Michał Budzik, Elżbieta Cipora, Izabela Gąska, Lucyna Gazdowicz, Aneta Mielnik, Katarzyna Sygit, Marian Sygit, Edyta Krzych-Fałta, Daria Schneider-Matyka, Szymon Grochans, Anna M. Cybulska, Jarosław Drobnik, Ewa Bandurska, Weronika Ciećko, Piotr Ratajczak, Karolina Kamecka, Michał Marczak, Remigiusz Kozłowski

**Affiliations:** 1 Department of Health Economics and Medical Law, Medical University of Warsaw, 01-445 Warsaw, Poland; 2 Department of Economic and System Analyses, National Institute of Public Health NIH-National Research Institute, 00-791 Warsaw, Poland; 3 Department of Oncology, The National Institute of Medicine of the Ministry of Interior and Administration, 02-507 Warsaw, Poland; 4 Department of Oncology Propaedeutics, Medical University of Warsaw, 01-445 Warsaw, Poland; 5 Medical Institute, Jan Grodek State University in Sanok, 38-500 Sanok, Poland; 6 Faculty of Health Sciences, Calisia University, 62-800 Kalisz, Poland; 7 Department of Basic of Nursing, Faculty of Health Sciences, Medical University of Warsaw, 01-445 Warsaw, Poland; 8 Department of Nursing, Faculty of Health Sciences, Pomeranian Medical University in Szczecin, 71-210 Szczecin, Poland; 9 Department of Specialised Nursing, Faculty of Health Sciences, Pomeranian Medical University in Szczecin, 71-210 Szczecin, Poland; 10 Department of Family Medicine, Faculty of Medicine, Wrocław Medical University, 51-141 Wrocław, Poland; 11 Center for Competence Development, Integrated Care and e-Health, Medical University of Gdansk, 80-204 Gdansk, Poland; 12 Department of Pharmacoeconomics and Social Pharmacy, Poznan University of Medical Sciences, 60-806 Poznań, Poland; 13 Department of Management and Logistics in Healthcare, Medical University of Lodz, 90-131 Lodz, Poland; 14 Collegium Management, WSB Merito University in Warsaw, 03-204 Warszawa, Poland

**Keywords:** pancreatic cancer, epidemiology, risk factors, screening

## Abstract

**Simple Summary:**

Pancreatic cancer is becoming a growing health problem. Its share in the proportion of neoplasms is increasing. Due to its asymptomatic course, this cancer is often diagnosed at a late stage, which makes treatment difficult. Diagnosis of pancreatic cancer is based on imaging tests, which have varying degrees of effectiveness. Additionally, these methods are costly. For these reasons, the implementation of population screening programs is not recommended. However, there are recommendations for screening people from high-risk groups.

**Abstract:**

Pancreatic cancer is the seventh most common cause of death in the group of oncological diseases. Due to the asymptomatic course, early diagnosis is difficult. Currently, early detection methods are only used in high-risk groups. A literature review based on the available results of observational studies on patients with pancreatic cancer and people from high-risk groups was used to summarize the knowledge on risk factors. The GLOBOCAN 2020 data were used to assess the epidemiological situation in Europe. A summary of screening recommendations was prepared based on the available documents from medical organizations and associations. Pancreatic cancer risk factors are divided into two main groups: non-modifiable factors, e.g., hereditary factors and age, which increase the risk of developing this disease, and modifiable factors—BMI, smoking, and alcohol consumption. Hereditary factors account for 10% of pancreatic cancer cases. The highly specialized methods of early detection, (MRI, CT, or EUS) are used for screening high-risk populations. Of all the imaging methods, EUS is considered the most sensitive for pancreatic cancer and allows an accurate assessment of the size of even small lesions (<30 mm) and the extent of tumour infiltration into blood vessels. The available studies vary on the level of sensitivity and specificity of these methods for the diagnosis of pancreatic cancer. EUS, MRI, and CT are also expensive procedures and in some patients can be invasive, which is one of the arguments against the introduction of population screening programs based on imaging methods. Therefore, it is important to look for viable solutions that would improve early detection. This is important from the point of view of healthcare systems in Europe, where almost 29% of all global pancreatic cancer cases are reported.

## 1. Introduction

There is a growing share of pancreatic cancer in the overall cancer incidence. Globally, pancreatic cancer is the seventh most common cause of death in the group of oncological diseases. Adenocarcinoma of the pancreatic duct is diagnosed most often—in over 85% of all patients. The high mortality rate and the low 5-year survival rate result from several reasons [1]. In most cases, cancer develops asymptomatically, which makes diagnosis difficult. The symptoms are often nonspecific, with most patients reporting pain, mainly dorsal, nausea, weight loss, and loss of appetite. For this reason, most cases are diagnosed at an advanced stage of the disease. Effective treatment is complicated by the fact that the only method that increases the chance of recovery is cancer resection at an early stage of its development [1,2]. Pancreatic cancer treatment methods depend on clinical staging and include surgical resection and chemotherapy as an adjuvant or neoadjuvant approaches for resectable and borderline resectable tumours, respectively; radiochemotherapy for unresectable, locally advanced disease; and chemotherapy and molecular targeted agents for metastatic cancer [3].

Due to the asymptomatic course, early diagnosis is complicated. It is currently assumed that early detection methods can only be used in high-risk groups. The CA 19-9 marker, whose concentration increases in neoplastic diseases, is most often determined from blood serum. However, the test sensitivity in the case of pancreatic cancer does not exceed 75%, while the test specificity is at most 85% [4,5]. These results do not allow for a precise distinction between cancer and other non-oncological diseases of the pancreas, as well as the liver and intestines. The methods that allow for the detection of a neoplastic lesion at a sufficiently early stage allowing for full resection are imaging methods—magnetic resonance imaging (MRI), endoscopic ultrasonography (EUS), or computed tomography (CT). These tests are not used in the general population but may be used in high-risk groups. People with a family history of pancreatic cancer, Peutz–Jeghers syndrome, Lynch syndrome, familial adenomatous polyposis, or ataxia–telangiectasia (AT) are particularly at risk. Additionally, the diagnostic methods used are expensive from the perspective of the healthcare system, which limits the possibility of their wider use in population screening programs [4,5,6].

The objective of this review is to present the current and future trends in the incidence of pancreatic cancer in Europe and to present the current recommendations for the early detection of pancreatic cancer. Based on the available studies on populations with pancreatic cancer and people from high-risk groups, a summary of the state of our knowledge on pancreatic cancer risk factors, broken down into modifiable and genetic factors, was made. The publications included in the work are indexed in the PubMed, Scopus, and ScienceDirect scientific databases. The publications were obtained based on searches containing the keyword combinations “epidemiology”, “pancreatic cancer”, and “risk factors”.

The GLOBOCAN 2020 (Global Cancer Observatory) data were used to assess the epidemiological situation in Europe. The GLOBOCAN is a specialized cancer agency of the World Health Organization. The GCO database contains data on 36 cancers by age and sex. The data come from 185 countries and the national cancer databases. Cancers in the database have been classified according to the international classification of diseases ICD-10 [7]. The time trends of the main epidemiological indicators discussed—incidence and mortality—for 5-year time intervals and by sex were presented. A summary of the screening recommendations was based on the available documents from medical organizations and associations with expert knowledge, operating at the national and international levels.

## 2. Pancreatic Cancer Risk Factors

Pancreatic cancer risk factors are divided into two main groups: non-modifiable factors, e.g., hereditary factors and age which increase the risk of developing the disease, and modifiable factors—BMI, smoking, and alcohol consumption.

Hereditary factors account for 10% of pancreatic cancer cases [6]. People whose relatives have developed pancreatic cancer are at an increased risk. The risk of developing the disease is 9-fold higher in the case of at least one first-degree relative with pancreatic cancer and increases 32-fold if at least three first-degree relatives have been diagnosed with this cancer [6,8]. In addition, having a relative that developed pancreatic cancer at an early age (<50 years old) increases the relative risk by 9.3 times [9]. Non-modifiable factors include the hereditary breast and ovarian cancer syndrome caused by mutations in the BRCA2 gene; the relative risk for BRCA2-mutant patients is 3 to 4-fold higher [10]. Peutz–Jeghers syndrome (PJS), inherited in an autosomal dominant manner, results from the mutation of the STK11/LKB1 gene and manifests as polyposis of the hamartoma type in the gastrointestinal tract and other organs; it increases the relative risk of pancreatic cancer up to 132 times, which is the highest risk among the genetic factors [11]. In the case of hereditary pancreatitis (HP), mutations in the PRSS1 gene appear in 80% of diagnoses, and the risk of developing pancreatic cancer compared to the general population in people with HP is high (RR = 69). Another syndrome with genetic changes that may favour the development of pancreatic cancer is familial atypical multiple melanoma syndrome (FAMMM), which in 38% of cases is caused by mutations in the P16INK4A/CDKN2A gene. This syndrome increases the risk of developing pancreatic cancer up to 22 times (RR = 13–22) [6,10,12].

Other non-modifiable, non-genetic risk factors include age and sex. According to the data, the incidence of pancreatic cancer globally was higher in men (5.7/100,000) than in women (4.1/100,000) in 2020 [7]. The mechanisms influencing the sex differences are not known; they might be related to lifestyle and environmental factors, e.g., increased risk when exposed to nickel at work [13,14]. Pancreatic cancer is most often diagnosed in people over 50 years of age, with the peak incidence in the 70–80 age range. Pancreatic cancer develops less often in people under 40 years of age and is usually associated with genetic factors. A diagnosis at an older age may be associated with an extended period of development of the disease before lesions in the organ undergo malignant progression [15].

The identified modifiable risk factors include smoking, improper nutrition leading to overweight and obesity, and low physical activity, which, in turn, are factors for type 2 diabetes [15,16]. In the European region, the WHO reported overweight or obesity in 59% of adults and almost 30% of children. The incidence of obesity in Europe is one of the highest in the world [17]. According to studies, obesity is particularly associated with pancreatic ductal adenocarcinoma (PDAC) [16]. Some of the studies focusing on a more detailed understanding of the relationship mechanism suggest the involvement of metabolic disorders in the metabolism of cytokines and signal receptors in the visceral adipose tissue and in the carcinogenesis of the organ [18]. It is estimated that the risk of developing pancreatic cancer increases by 10% or more for every 5 kg/m^2^, or by 20–50% in people who are already obese [19]. Participants in the Arslan et al. study who were obese, were diagnosed with pancreatic cancer on average one year earlier compared to normal-weight participants [20]. Another study conducted by Genkinger at al. indicated that an increase in BMI over 10 kg/m^2^ in adults increased the risk of pancreatic cancer 1.4 times compared to the group whose BMI increased by no more than 2 kg/m^2^ [21]. The results of a case–control study conducted by Li et al. showed that with the median incidence at the age of 64, obesity at the age of 20–49 translated into an earlier onset of the disease by two to six years [22]. Obesity and high BMI have been linked to hyperglycaemia, insulin resistance, and diabetes, factors that are also linked to pancreatic cancer. Aberrant signal transduction by insulin growth factor can potentially, through the mechanism of apoptosis and cell proliferation, affect the promotion and development of cancer [23]. The mechanisms linking type 2 diabetes (T2DM) with pancreatic cancer are complex [24,25]. According to research, approx. 80% of people suffering from pancreatic cancer have insulin resistance or developed diabetes. The incidence of cancer in patients with type 2 diabetes (T2DM) increases by 10% compared to the general population, and long-term diabetes is associated with a 1.5–2.0-fold increase in the risk of pancreatic cancer [26]. The results also indicate that diabetics who developed the disease at > 50 years of age, accompanied by weight loss and damage to the exocrine system, are at a higher risk of pancreatic cancer [27]. Smoking tobacco products is a confirmed risk factor for many cancers, including pancreatic cancer. Korc et al. in a summary of ten meta-analyses found that the combined relative risk was higher in the group of smokers compared to non-smokers (RR = 1.66; 95% CI 1.38–1.98) [28]. In the study conducted by Bosetti et al., the mean odds ratio for active smokers was 2.20 (95% CI 1.71–2.93) compared to non-smokers. The odds of developing the disease decreased with the reduction in smoking: the OR of former smokers was 1.17 (95% CI 1.02–1.34) compared to non-smokers [29]. Tranah et al. calculated that for non-smokers > 25 years of age the OR was 0.98 (95% CI 0.72–1.3), 15 < 20 years of age OR = 0.84 (95% CI 0.5–1.4), and for those who stopped smoking in less than 10 years OR = 1.6 (95% CI 1.1–2.3) [30]. It is also assumed that passive smoking may also be a risk factor for pancreatic as well as other cancers [31].

## 3. Pancreatic Cancer Epidemiology

Globally, among cancers, lung cancer remains the leading cause of death with over 1.7 million deaths annually. Colorectal cancer ranks second with 935,173 deaths, while pancreatic cancer ranks seventh with 466,003 cases worldwide. Across Europe in 2020, pancreatic cancer was responsible for 132,134 deaths, and the annual number of new cases was 140,116. As a result, in Europe, almost 29% of all deaths are due to this cancer. The risk of developing pancreatic cancer in Europe is high—2.31/100,000 of the population. It is thought that the significant share of European countries in the global incidence of pancreatic cancer is not only the result of share of risk factors but also because of better developed healthcare systems, and thus diagnostics that allow for the detection of a greater number of cases and better determination of the cause of death (Table 1) [32].

**Figure 1:**
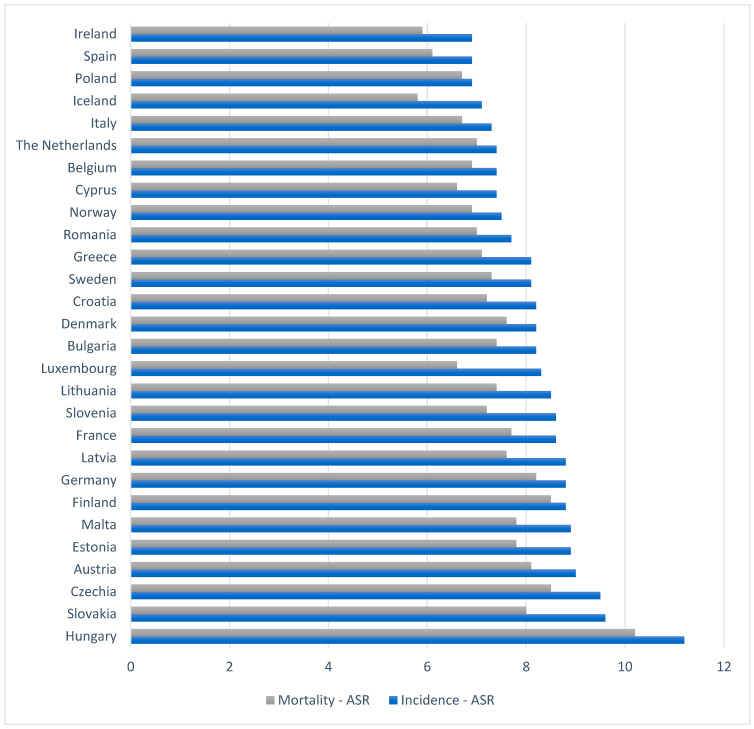
Age-standardized 5-year incidence and mortality rates in select European countries in 2020 [7].

Figure 1 shows the 5-year age-standardized values of pancreatic cancer incidence and mortality rates in Europe. In 2020, the highest incidence rate among all EU countries was recorded in Hungary (11.2/100,000), Slovakia (9.6/100,000), and the Czech Republic (9.5/100,000). Hungary was also the country with the highest mortality rate for pancreatic cancer, with the Czech Republic in second place with a rate of 8.5/100,000. The lowest rates were recorded in Ireland (incidence 6.9/100,000 and mortality 5.9/100,000) and Spain, where the 5-year incidence rate was 6.9 and the mortality rate was 6.1. The incidence of pancreatic cancer was higher in men than in women (9.4 vs. 6.4). This translates into higher mortality rates for men (8.8 for men and 5.8 for women) [32].

**Figure 2:**
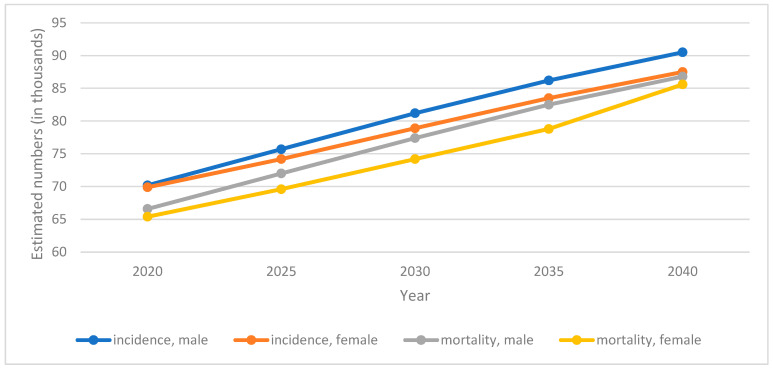
Projection of pancreatic cancer incidence and mortality in Europe by sex [7].

Predictions show that the number of cases of pancreatic cancer will increase. By 2025, it is estimated that the number of new cases will increase by 9829, and by 2040 by 37,824, which will give 177,940 cases. Mortality will increase to 141,653 deaths in 2025, and to 169,389 in 2040 (37,255 more deaths than in 2020) (Figure 2). In the breakdown by sex, the predominance of men was observed for both indicators.

## 4. Pancreatic Cancer Screening Recommendations

Due to its asymptomatic course, pancreatic cancer is difficult to diagnose. In diagnosing pancreatic cancer, a combination of blood biomarker tests and imaging tests is used. Carbohydrate antigen 19-9 (CA 19-9) determined from the serum is used to detect gastrointestinal cancers and is currently the most used biomarker for pancreatic cancer. In patients with pancreatic cancer with abnormal concentrations of the marker, CA 19-9 correlates with the stage of cancer or the tumour size; therefore, it can be used to assess the prognosis and the possibility of surgical removal of the tumour [33,34]. In the interpretation of the results, a concentration of CA 19-9 above 500/mL in most cases results from the neoplastic process. This marker alone cannot be treated as proof of the presence or absence of cancer due to insufficient sensitivity and specificity. In the screening of pancreatic cancer, the main imaging tests used include endoscopic ultrasonography (EUS), magnetic resonance imaging (MRI), and computed tomography (CT) with pancreatic protocols. In addition, abdominal ultrasonography (US), magnetic resonance cholangiopancreatography (MRCP), and positron emission tomography (PET) are performed [34]. The use of a single imaging test is not recommended for the assessment of the preoperative stage of pancreatic cancer, as indicated in the study by Soriano et al. [35]. According to their results, computed tomography allows for the most accurate assessment of tumour extent, metastases, and staging. In addition to CT, EUS is considered to be the most sensitive method for imaging pancreatic cancer and allows for accurate assessment of the tumour size and the degree of infiltration into blood vessels [33,34]. The median sensitivity of EUS in the study conducted by Yoshida et al. in detecting lesions in the pancreas was 93–94%, the sensitivity of MRI was 67%, and that of CT was 53% (for detecting lesions <30 mm, n = 49). The advantage of the EUS method is its ability to detect small lesions in the pancreas, below 30 mm [36,37]. On the other hand, MRI/MRCP shows higher sensitivity to cystic lesions of diverse sizes [38]. Rhee et al. summarized the results of the diagnostic efficiency of the imaging methods used to detect pancreatic cancer and their strengths and weaknesses [39]. EUS sensitivity was estimated at 89–91%, with a specificity of 81–86%; the main advantages of this method is its high resolution and the ability to combine it with needle biopsy, while the disadvantage was the invasive nature of the examination. In Rhee’s review, based on results from meta-analyses, the MRI method had a sensitivity of 84–93%, and specificity of 82–89%, which are higher than those in Yoshida’s study. The advantage of the MRI method was a good visualization of pancreatic and biliary abnormalities, while the disadvantage was a lower spatial resolution. Computed tomography achieved a sensitivity of 89–91% and a specificity of 85–90% with high temporal and spatial resolution and limited effectiveness with small lesions in the pancreas, liver, and peritoneum. In addition, the use of the ultrasonography was evaluated—this method can be used in the initial assessment of lesions in the pancreas. Its sensitivity was estimated at 68–95% and specificity at 50–100% [39].

Population screening for pancreatic cancer is currently not recommended. The US Preventive Services Task Force (UPSTF) gave a negative opinion on this issue. There is no evidence that early detection and treatment of pancreatic cancer improves morbidity or mortality rates. The low incidence of pancreatic cancer compared to other malignancies in the population, poor prognosis even at an early stages, the accuracy of testing methods and their high cost translate into low effectiveness and health benefits of a possible population-based programme [40]. However, there are recommendations for screening people at high risk. The goal of monitoring people at high risk for pancreatic cancer is to detect precursors or pancreatic cancer at a stage that allows resection. The aim of these activities is primarily to improve the quality of life of patients and reduce cancer complications [41]. The American Gastroenterological Association (AGA) has developed a set of thirteen good practice guidelines for screening people at high risk. The AGA recommends screening people over 50 years of age or 10 years younger (if there was a family history of the disease) than the age of cancer diagnosis in a relative. The recommended methods of testing include MRI and EUS, used in combination. In addition, individuals over 40 years old should be screened if they carry the CKDN2A and PRSS1 mutations in combination with hereditary pancreatitis, as well as people over age of 35 who have been diagnosed with Peutz–Jeghers syndrome. In the absence of organ lesions, the AGA recommends the introduction of annual intervals in testing, and shortening the time interval if low-risk lesions are detected. It is recommended to perform EUS within 3–6 months for indeterminate lesions and within 3 months for high-risk lesions if no resection is planned [42]. The American Society for Gastrointestinal Endoscopy recommends screening individuals with an increased risk of pancreatic cancer due to genetic susceptibility, including BRCA2 and BRCA1 gene mutations, using imaging methods—EUS, MRI, or a combination of both methods. The ASGA does not provide specific age ranges for inclusion due to the low quality of scientific evidence [43]. The same recommendations are provided by Cancer Research UK, according to which, screening should involve p high-risk individuals with hereditary pancreatitis and a defect in the PRSS1 gene, Peutz–Jeghers syndrome, or first-degree relatives with pancreatic cancer and those with a mutation in the BRCA1, BRCA2, PALB2, or CDKN2A genes. The organization also recommends the use of MRI or MRCP, EUS, or CT in diagnostics [44]. The recommendations of the National Institute of Health and Care Excellence (NICE) additionally include considering monitoring individuals with two or more cases of a first-degree relative diagnosed with pancreatic cancer, within two or more generations. As a diagnostic test, the NICE recommends MRI/MRCP or EUS in people without hereditary pancreatitis and CT in those with hereditary pancreatitis and a PRSS1 mutation [45]. The International Cancer of the Pancreas Screening (CAPS) Consortium recommends considering screening people with more than one relative diagnosed with pancreatic cancer, particularly if the relative was diagnosed before the age of 50. The risk of developing pancreatic cancer depends on the number of affected relatives and their connections. Screening should be conducted at 12-month intervals in high-risk groups and every 3–6 months in cases of newly diagnosed pancreatic cancer [46] (Table 2).

The effectiveness of the existing pancreatic cancer screening has been evaluated by some researchers. In the CAPS5 (Cancer of Pancreas Screening-5) multicentre study, 26 cases of pancreatic cancer were found in 1731 people included in the study; 57.9% of the diagnoses were early-stage diagnoses [46]. The median survival of patients under CAPS follow-up was 9.8 years, and the 5-year survival rate was 73.3%. Dbook et al. compared this data with the results of unsupervised patients, where the median survival was 1.5 years, and the majority of cases were diagnosed at an advanced stage of the disease. It can be concluded that current screening protocols may be helpful in recognizing more patients at the onset of the disease and contribute to increased survival rates [47]. The results of a systematic review by Lu et al. also indicate similar findings: screening of patients with familial pancreatic cancer was associated with a higher rate of cancer detection at an early stage and increased patient survival [48].

## 5. Conclusions

Pancreatic cancer is becoming a growing health problem. Its high mortality due to the asymptomatic course in the early phase and poor prognosis for diagnosed patients make pancreatic cancer an increasing challenge for health care systems. According to projections, the share of pancreatic cancer in the incidence of all cancers will increase, and with our current knowledge, medicine does not have an effective method for treating pancreatic cancer. The highly specialized methods currently used for early detection, such as MRI or EUS, are expensive and can be invasive, which is one of the arguments against the introduction of screening programs. Therefore, it is important to look for viable solutions that would improve early detection. This is important from the point of view of healthcare systems in Europe, where almost 29% of all pancreatic cancer cases worldwide are reported. In recent years, some attention has been drawn to the potential use of artificial intelligence and deep learning using false neural systems in early detection based on data from social media, or the use of these methods in abdominal imaging. The practical use of these tools requires further in-depth research.

## Figures and Tables

**Table 1:** Incidence and mortality of pancreatic cancer in the world and in Europe in 2020.

		World	Europe
Incidence	Number (95% UIs)	495,773 (488,953.0–502,688.0)	140,116 (136,953.0–143,352.0)
	Crude rate ^a^	6.4	18.7
	ASR ^a^	4.9	7.8
	Cumulative risk ^b^	1.63	2.31
Mortality	Number (95% UIs)	466,003 (459,505.0–472,593.0)	132,134 (129,563.0–134,756.0)
	Crude rate ^a^	6.0	17.6
	ASR ^a^	4.5	7.2
	Cumulative risk ^b^	1.58	2.21

^a^—per 100,000 population. ASR—age standardized rate. ^b^—in %, population aged 0–85+ years [7].

**Table 2:** Characteristics of pancreatic cancer screening guidelines based on literature reviews from expert organizations—examples.

Institution	Genetic Factors	Medical History	Testing Method	Time Pattern of Screening
**International Cancer of the Pancreas Screening (CAPS) Consortium**	BRCA1, BRCA2, PALB2 gene mutation (age 45–50); Peutz–Jeghers syndrome (age 40); FAMMM syndrome; hereditary pancreatitis with a mutation in the PRSS1 gene (age 40); HNPCC syndrome; ataxia–telangiectasia syndrome	>1 first-degree relative with pancreatic cancer; 1 first-degree relative and 1 second/third-degree relative; >2 second-degree relatives; >3 relatives with pancreatic cancer, regardless of the degree of relationship	MRI/MRCP + EUS + fasting glucose and/or HbAlc; MRI/MRCP or EUS during follow-up examination; CT for a solid tumour or asymptomatic stenosis of the pancreatic duct of unknown aetiology	12-month intervals when there are no concerning pancreas lesions; 3 or 6 months if concerning abnormalities for which immediate surgery is not indicated (solid or cystic lesion size ≤ 5 mm, MDP dilation ≤ 10 mm)
**American Society for Gastrointestinal Endoscopy (ASGE)**	BRCA1, BRCA2, PALB2 gene mutation (age 50 ^a^ or 10 years less ^b^); FAMMM syndrome (age 40 ^a^ or 10 years less ^b^); Peutz–Jeghers syndrome (age 35 ^a^ or 10 years less ^b^); Lynch syndrome (age 50 ^a^ or 10 years less ^b^); hereditary pancreatitis (age 40 ^a^)	at least 1 first-degree relative with pancreatic cancer (age 50 ^a^ or 10 years earlier ^b^);	EUS preferred in people from very high risk groups (with Peutz–Jeghers syndrome, FAMMM syndrome) ^c^ EUS + panendoscopy/colonoscopy—Lynch syndrome, Peutz–Jeghers syndrome ^c^; MRI—in people at increased risk of an adverse event related to anaesthesia or invasiveness of the examination	Annual screening to be performed
**American Gastroenterological Association**	BRCA1, BRCA2, PALB2, ATM gene mutation (age 50 ^a^ or 10 years less ^b^); Peutz–Jeghers syndrome (age 35 ^a^); hereditary pancreatitis with a mutation in the PRSS1 gene (age 40 ^a^); FAMMM syndrome (age 40 ^a^); Lynch syndrome	>2 first-degree relatives who do not meet the criteria for other hereditary cancer syndromes	EUS, MRI, EUS + MRI	12-month intervals when there are no concerning pancreas lesions; In low risk lesions, EUS every 6-12 months; For indeterminate lesions, EUS every 3–6 months; 3-month interval for high-risk lesions (if resection is not planned)

FAMMM—familial melanoma-associated dysplastic nevus syndrome, HNPCC—hereditary non-polyposis colorectal cancer syndrome; EUS—endoscopic ultrasonography, MRI—magnetic resonance imaging, MRCP—bile duct magnetic resonance imaging, CT—computed tomography. ^a^—if a lesion is present, the recommended age to start screening. ^b^—the age at which screening is commenced is lowered to 10 years earlier than the youngest age that a family member was diagnosed with pancreatic cancer. ^c^—recommended when MRI cannot be performed due to claustrophobia, contrast allergy, metal implants, kidney failure, lack of patient consent to an invasive method [40,42,43,45].

## Data Availability

Data available from authors.
